# The onset and progression of alcohol use disorders: A qualitative study from Goa, India

**DOI:** 10.1080/15332640.2017.1326863

**Published:** 2017-06-30

**Authors:** Nathalie Mackinnon, Urvita Bhatia, Abhijit Nadkarni

**Affiliations:** 1 McGill University, Montreal, Canada; 2 Addictions Research Group, Sangath, Goa, India; 3 London School of Hygiene and Tropical Medicine, London, United Kingdom

**Keywords:** Alcohol use disorders, India, onset, progression, qualitative

## Abstract

Quantitative evidence about the burden of alcohol use disorders (AUDs) needs to be complemented with a nuanced qualitative understanding of explanatory models to help supplement public health strategies that are too often steeped uncritically in biomedical models. The aim of this study was to identify the role of various factors in the onset and persistence of AUD and recovery from AUD. This was a qualitative study nested in a population cohort from Goa, India. In-depth interviews of men with incident, recovered, and persistent AUD covered topics such as changes in drinking habits over time, perceptions and experiences about starting/stopping drinking, and so on. Data were analyzed using thematic analysis. Reasons to begin drinking included social drinking, functional use of alcohol, stress, and boredom. Progression to problematic drinking patterns was characterized by drinking alone, alternating between abstinent and heavy drinking periods, and drinking based on the availability of finances. Some enablers to reduce/stop drinking included consequences of drinking lifestyle and personal resolve; some barriers included availability of alcohol at social events and stress. Some reasons for persisting heavy use of alcohol included lack of family support, physical withdrawal symptoms, peer pressure, stress, and easy availability. This article offers a strong conceptualization and nuanced understanding of AUD across a spectrum of developmental courses. This adds to the limited literature on explanatory models of AUD in India and identifies potential targets for prevention and treatment strategies for AUD in low- and middle-income country settings.

## Introduction

Alcohol use disorders (AUDs) are a growing cause for concern worldwide, accounting for 5% of the total global burden of disease (World Health Organization, 2014). This burden is growing disproportionately in low- and middle-income countries (LMIC), where rising disposable income, urbanization, changing social norms, and aggressive marketing of alcohol contribute to problematic drinking patterns (Benegal, 2005; World Health Organization, 2014). India has one of the largest and fastest-growing AUD-attributable burdens globally, and almost half the drinkers in India exhibit hazardous drinking patterns (Benegal, 2005; Rehm et al., 2009).

The longitudinal progression of AUD has been studied extensively in developed countries (Finney & Moos, 1991; Gual, Lligona, & Colom, 1999; O’Connor & Daly, 1985), but such evidence from India is limited by small sample sizes, short follow-up periods, and so on (Kar, Sengupta, Sharma, & Rao, 2003; Kuruvilla & Jacob, 2007; Mohan, Chopra, & Sethi, 2002). In a follow-up study of a community cohort of men, Nadkarni, et al. (2016) found that 15.0% of casual drinkers had developed AUD, and 22% of those with AUD had stopped drinking at 6-year follow-up (Nadkarni et al., 2016). Although such quantitative evidence is crucial, it needs to be complemented with a nuanced qualitative understanding of why people develop AUD or recover from it. Such an understanding can help supplement public health strategies that are too often steeped uncritically in biomedical models (Napolitano & Jones, 2006) devoid of the explanatory models held by those with AUD. Furthermore, all understandings of health and illness incorporate cultural elements (Kleinman, 1980), and hence a patient’s psychosocial view of illness might contrast with the biomedical perspective of disease (Eisenberg, 1977). Understanding such differences (or similarities) provides vital information for the optimal design of interventions, helps bridge sociocultural differences between health care providers and patients (Hahn & Inhorn, 2009), promotes empathy and therapeutic alliance, corrects the overemphasis on biology in the biopsychosocial model, and elaborates on the cultural context and meaning of varying notions of illness in a globalizing world (Weiss & Somma, 2007).

The aim of this study was to fill the gap in literature by examining the onset and progression of AUDs from the perspective of those with AUD. The specific objectives of this study were to identify the role of various factors in the (a) onset and persistence of AUD and (b) recovery from AUD.

## Methods

### Study design

This was a qualitative study nested in a population cohort.

### Setting

The study was conducted in the state of Goa, one of India’s smallest states, with a population of just over 1.4 million people (Government of India, 2011). Goa ranks fourth in the National Human Development Index, with one of the lowest poverty indicators in the country (Gandhi et al., 2011). Alcoholic drinks are easily available here at cheaper rates than in neighboring states due to lower excise duties (Patel, Dourado, De Souza, & Dias Saxena, 2001) and local production of alcohol from the cashew fruit.

### Sample

From 2006 to 2008, a cross-sectional survey (1,899 men, aged 18–49 years) was conducted in urban and rural areas of Northern Goa (Pillai et al., 2013). A two-stage probability sampling procedure, based on electoral rolls, was used to select the population-based sample. From a randomly selected household, the participants were selected at random from those of eligible ages within the households. Refusal rates for randomly selected households were 1.5%. In 2012–2014, all these men were approached again to measure a range of outcomes at follow-up (Nadkarni et al., 2016). Along with a range of other data, information about AUD was collected by administering the 10-item Alcohol Use Disorders Identification Test (AUDIT) (Saunders, Aasland, Babor, DeLaFuente, & Grant, 1993) at baseline and follow-up. AUD was diagnosed using an AUDIT cutoff score of ≥8 and hence included hazardous, harmful, and dependent drinkers. We created a sampling frame with the following categories: incident AUD (no AUD at baseline, AUD at follow-up), recovered AUD (AUD at baseline, no AUD at follow-up), and persistent AUD (AUD at both baseline and follow-up). Within each of these categories, we selected participants using maximum variation sampling to allow for representativeness of a wide range of parameters, for example, age and employment status. Maximum variation sampling is a type of purposive sampling strategy that aims for representativeness by including a wide range of extremes rather than through equal probabilities. The advantage of such sampling is that it allows a richer and more authentic representation of the phenomenon under study, although sometimes at the risk of reduced generalizability because of extreme diversity in responses.

### Data collection

Data were collected using in-depth interviews (IDIs), a technique that allows for detailed in-depth probing of subject matter and provides information on context (Legard, Keegan, & Ward, 2003). Sociodemographic data were collected, and the IDI covered topics such as major changes in participants’ lives since baseline, changes in drinking habits over time, perceptions and experiences about starting/stopping drinking, and any treatment sought. Research workers trained in qualitative data collection conducted the IDIs in the vernacular.

### Data management and analyses

Audio-recorded interviews were transcribed verbatim and then translated into English. Data were analyzed by NM under supervision of AN and UB. Thematic analysis, a method for identifying, analyzing, and reporting patterns (themes) within data (Braun & Clarke, 2006) was used to analyze the data. Initially, analysis involved inductive generation of new codes from analyzing the raw data from a few transcripts. These codes were then used to generate a new coding template, which was then applied to the remaining interviews. This coding template served as a framework for analyzing and organizing subsequent data. NM read the transcripts to immerse herself in the data and then applied the codes from the template. Themes were derived by retrieving pieces of data pertaining to codes and by examining their meaning in relation to the research questions. Patterns were derived by comparing similarities and differences between participants on these themes or by examining how the themes or codes were connected to or interacted with one another. Each theme was assigned a name and a descriptive phrase that best explained its meaning and united its individual codes on consistency. The themes were supported by excerpts from transcripts to demonstrate that themes were as close to the data as possible and reflected the words used by the participants themselves.

### Ethics

Ethical approval for the qualitative study was obtained from the Sangath Institutional Review Board (IRB). Written informed consent was taken individually from all participants, and permission to record the interview sessions on the digital audio recorder was sought from each participant prior to the interview.

## Results

We interviewed 20 men with persistent AUD, 19 who had recovered from AUD, and 23 who had incident AUD. Table 1 describes the sociodemographic profile of the participants. The mean age of the participants was 42 years. Compared to those with incident and persistent AUD, a larger proportion of those with recovered AUD lived in urban areas. Compared to those with persistent AUD, a larger proportion of those with incident and recovered AUD were married and employed. A higher proportion of those with recovered and persistent AUD were literate as compared to those with incident AUD. Mean AUDIT score at baseline was higher in those with persistent and recovered AUD, compared to those with incident AUD. On the other hand, mean AUDIT score at follow-up was higher in those with persistent and incident AUD compared to those with recovered AUD. There was not much difference in the age of onset of drinking among the three AUD trajectories.

**Table 1.** Sociodemographic profile and drinking patterns of the participants.

	Incident AUD *N* = 23 *n* (%)	Persistent AUD *N* = 20 *n* (%)	Recovered AUD *N* = 19 *n* (%)	Total *N* = 62 *n* (%)
Mean age in years (*SD*)	42 (9.2)	42.7 (6.9)	41.8 (8.1)	41.8 (8.6)
Urban residence	5 (21.7)	7 (35.0)	10 (52.6)	22 (35.5)
Employed	20 (87.0)	13 (65.0)	18 (94.7)	51 (82.3)
Married	20 (87.0)	13 (65.0)	17 (89.5)	50 (80.7)
Literate	19 (82.6)	19 (95.0)	18 (94.7)	56 (90.3)
Mean AUDIT at baseline (*SD*)	1.3 (2.2)	15.1 (7.3)	15.8 (6.3)	10.2 (8.8)
Mean AUDIT at follow-up (*SD*)	11.9 (5.7)	18.2 (5.0)	0 (0)	10.3 (8.6)
Mean age at onset of drinking (*SD*)	20.3 (4.8)	21.1 (4.8)	21.0 (4.8)	20.9 (4.7)

AUD = alcohol use disorder; *SD* = standard deviation.

### How do people develop AUD?

#### Onset of drinking

*Social drinking or drinking for pleasure*. The majority of users began drinking within social circles, with friends or coworkers, and did not view the drinking as problematic initially. Over time, the drinking became more frequent: “It was in 2007 that I started drinking. It happened for fun in the company of friends when I was working, and gradually increased” (Employed, 33 years). Although it was generally believed that it was their personal choice to drink, peer pressure was sometimes reported. “My friends forced me to drink and I used to drink ½ peg just to satisfy my friends” (Unemployed, 57 years).

*Functional use*. Users cited alcohol as a means to overcome pain or fatigue or to help cope with a specific task (e.g., driving over long stretches of time). One cook described how work-related fatigue contributed to his drinking:

I come home from work and then I work in our fast food restaurant … where I also help out as a cook … and then due to work load and fatigue I have a beer … this happens almost every day. (Employed, 31 years)

*Stress*. Psychological stresses, often described as “tensions,” were instigators of problematic drinking patterns. Participants cited financial, familial, and work problems as sources of stress for which they self-medicated with alcohol.

It was due to the tensions I was facing then, I started drinking and now it has continued. I was in debt due to failure in my business. My financial state was bad. It was difficult for me to face the lenders and with all this unbearable tensions I started drinking (Employed, 32 years)

*Boredom*. In rural communities, being idle or staying at home all day were cited as justifications for drinking. The drinking was attributed to boredom, excessive free time, and the combined effect of idleness and unemployment. “I drink when I am not doing anything in particular. I don’t drink otherwise” (Employed, 40 years).

#### Progression of drinking

The progression of casual drinking to more problematic drinking patterns was characterized by drinking alone, alternating between abstinent and heavy drinking periods, and drinking based on the availability of finances.

*Lone drinking*. While drinking started initially with friends, progression to lone drinking was a common occurrence in later stages of their drinking: “Initially I went (to drink) with my friends. As I got used to it I went alone” (Employed, 33 years).

*On and off drinking pattern*. A common drinking pattern was stretches of abstinence between long periods of heavy drinking. Such periods of abstinence were cited as proof of nonaddiction, and problematic drinking was equated to daily heavy drinking.

I don’t drink every day. If I stop drinking I don’t drink for years. Like last time I had not drank for almost two years. So I can stay without drinking for one or two months. But if I drink, I drink continuously for four to eight days. (Part time work, 44 years)

*Drinking based on availability of finances*. The easy availability of funds was an enabler of more problematic drinking, while financial limitations in heavy drinkers tended to promote consumption of cheaper spirits. Rarely, participants reported drinking less rather than compromising on the quality of drinks when they were short on money. “We drink branded drinks like Scotch and whisky. If we have less money then we will reduce the quantity [of alcohol consumed], not the quality” (Employed, 53 years).

### Why do people with AUD stop/reduce their drinking?

#### Enablers

*Consequences of drinking lifestyle*. Those with AUD chose to stop drinking after they gained an understanding of the consequences of drinking on themselves, their peers, or their family.

I used to get the urge to drink, but I controlled this urge as I was concerned about my health. I was also concerned about my family and the problems they would have to face if anything happened to me. These concerns about my health and family made me quit drinking and it is the same till date. (Employed, 49 years)

In a few cases, individuals observed the negative behavior of their peers when drinking and were motivated to quit to avoid the same fate. “After seeing people suffer from drinking I decided to quit. A young man from my village and my brother-in-law expired due to excessive drinking” (Self-employed, 42 years).

A prominent theme that emerged was the decision to stop or reduce drinking following physical illness. People with AUD would experience either an alcohol-related or unrelated health problem and would stop drinking to avoid future health consequences. For example, after suffering an ophthalmological problem, one participant quit at the advice of his physician: “… since my doctor had informed me that if I drink, will have problem [eye would be affected] and because of that fear I stopped drinking” (Employed, 42 years).

*Personal resolve*. The majority of participants attributed their successful recovery to their personal strength and willpower to quit. Regardless of whether they received help from a family member or a doctor, many believed that the only means to a successful recovery was self-control.

After quitting alcohol I was free from all the problems which I was suffering such as giddiness, trembling, urge to drink more. Many people asked me about the deity I prayed to for quitting. I don’t know much about God, but it was my own will which helped me quit my drinking habit. (Self employed, 42 years)

*Support from family and friends*. Family support, either indirectly or directly, was consistently cited as an enabler of recovery. In many cases, the wife of the drinker would disapprove of her spouse’s drinking and encourage him to quit. In other cases, a family member would take direct action, acquiring medicine for the drinker or taking him to see a doctor.

#### Barriers

*Availability of alcohol at social events*. Participants who drank following a period of recovery frequently cited they drank because of a social event. Others were encouraged to drink by their peers at social events, although all did not always succumb to the pressure by their peers:

If I go out at night and meet someone [friends], they coax me to drink. That’s why I avoid going out at night. Last Narkasur [local festival], my friends were asking me to join them for drinks. I did not join them. I told them I have to go home and moved away from them. (Employed, 30 years)

*Functional use of alcohol*. In many cases, people returned to drinking as a relief from tiring work and to induce sleep. Drinking following work was a way to cope with either physical pain or mental exhaustion.

*Stress*. Family stress was considered a major barrier to recovery. Either family discord or stress to provide for their spouse caused participants to experience “tension,” which ultimately led to drinking as a coping mechanism.

#### Sources of help and coping strategies

*Self-help*. Many recovered participants attributed their success to self-help strategies, regardless of whether they sought external help or not. Three main techniques emerged from the interviews: replacement of alcohol with nonalcoholic alternatives (e.g., soft drinks), resisting thoughts of alcohol, and gradually reducing their intake.

*Spiritual or religious help*. An important source of support was informal help, most frequently from spiritual or religious sources. For example, one participant refrained from drinking alcohol one day a week in order to worship his god. More frequently, participants would stop drinking for an entire month during the Hindu holy month of Shravan.

*Formal help*. Formal help from a physician was frequently sought but not necessarily accredited with successful recovery. In many cases, a physical health problem instigated a visit to the doctor, most frequently leading to the prescription of medicines such as tablets, intravenous fluids, or simply advice to quit drinking. In such consultations, it was very rare for anything but the physical health issue to be discussed.

### Why people continue to have AUD

*Lack of family support*. In some cases, family members encouraged drinking behavior by simultaneously partaking, as in the case with one participant who drank with his brother:

Yes … I had stopped drinking … but when I used to bring liquor for him [brother] … I started drinking heavily again. (Unemployed, 36 years)

In many other cases, the families identified a problem but provided no support to the drinker to address it.

*Physical withdrawal symptoms*. In many cases, the physical withdrawal symptoms deterred participants from beginning or sustaining their recovery. In many cases, the participants were motivated to quit but could not overcome the negative consequences of withdrawal:

No, it [alcohol] does no good. I feel I should quit. But I cannot do so. Whenever I try to do it I cannot sleep and get hallucinations. (Self-employed, 40 years)

*Peer pressure*. Peer pressure was consistently mentioned as a barrier to recovery, and many participants mentioned the difficulty associated with hanging out with drinking friends.

Then all the men from my work place would go, there would be alcohol. I would say I have quit, they would force me to take a little beer. Once I drank then it would not stop. (Employed, 54 years)

*Stress*. Similar to those who developed AUD, those with persistent AUD also cited psychological stress as a barrier to recovery. These included psychological stress from day-to-day activities and other stressors that were more acute and actionable:

Not major tensions as such but the normal ups and downs of life sometimes make you drink. I have borrowed money which I have to repay, running of my business, also the minor issues in the family such as tensions between my mother and wife. All this gives you sleepless nights so to do away with it you tend to drink at times. (Self-employed, 30 years)

*Perception that drinking has not affected their life adversely/lack of understanding that health problems are affected by alcohol*. In many cases, there was a lack of awareness of the drinking problem and the belief that sobriety didn’t have substantial benefits.

*Beliefs about medications*. These included beliefs such as medications are the only solution, medications don’t work, or one cannot quit drinking without medications. In contrast to the recovered AUD participants who believed self-control was the solution to recovery, many persistent AUD participants believed that medicines were required to quit. They sought help from doctors and placed a heavy reliance on the ability of medicines to cure them.

But you require something [medications] for it … means you should feel like not drinking [after taking the medication] … feel drained or feel bad about it. If there is some remedy or some medicine for it then it can be tried. (Employed, 54 years)

*Idleness and boredom*. In a few cases, participants attributed their drinking to their boredom, similar to those who developed AUD. Many were at home due to an injury or unemployment and drank as a means to pass the time.

*Pain management*. Similar to those who developed AUD, a main barrier to recovery was the functional use of alcohol. For example, one participant who worked as a laborer complained of body aches after work:

I am a welder, so after the days hard work the body aches and it is difficult to get sleep. So I drink a little to do away with the pain. (Self employed, 30 years)

*Acceptance of drinking as an inevitable habit*. In many cases, participants recognized that they had drinking problems, but at the same time conceded that the habit was inevitable and they weren’t sure how to overcome it.

There is nothing good that I feel about alcohol … . But once you get addicted you can’t quit … and then there is this tension about the health, the frustration about bad health. (Employed, 54 years)

*Easy availability*. The availability, both financially and physically, was cited as a reason for continued drinking. Some had easy access because of work or neighborhood. One participant worked in a traditional alcohol manufacturing unit and therefore could drink for free, encouraging the behavior and reinforcing it later.

Yes … I needed more and more to get intoxicated … and I had money … my salary was 700 rupees … apart from that I got a daily allowance, liquor was cheap then … today the allowance is 100 rupees, but living costs have gone up … all the bar and tavern owners know me well … so they do allow a little credit. (Employed, 49 years)

## Discussion

Our study sought to examine the factors that contribute to the development and persistence of AUD and recovery from AUD. We found that the development of AUD was initially driven by social or functional reasons and progression was marked by drinking alone, drinking cheaper spirits to save money, and often drinking in binges. Recovery was impeded by lack of awareness about consequences of alcohol, unsupportive family environments, and the perceived need to drink alcohol to cope with stress. Conversely, those who recovered cited an awareness of the physical consequences and, less commonly, social consequences of drinking. Many cited poor physical health as an instigator to sobriety, and interestingly, despite consulting a doctor for their physical ailment, few attributed their reduction of drinking to any medical treatment. Instead, they attributed their success to personal resolve and self-control. Thus, strong self-will was the most poignant factor that was perceived to contribute to recovery. Notably, despite their success, participants still cited many barriers to recovery, such as presence of alcohol at social events and stress.

Many of our findings are consistent with global evidence. Existing evidence has attributed peer influences (Kuntsche et al., 2005; Yan et al., 2008), psychological stress (Brady & Sonne, 1999), and subjective pain relief (Brennan et al., 2005; Cutter et al., 1976) to the onset of AUDs. Studies from South Asia have attributed onset of AUDs to psychological causes (e.g., stress), sociocultural causes (e.g., fate), drinking for pleasure, and functional use (e.g., to alleviate pain) (Nadkarni et al., 2013a). The latter was considered the strongest risk factor for drinking among Sri Lankan male drinkers (Perera & Torabi, 2009), which is consistent with our current findings. Our results for coping strategies are consistent with the literature in that self-help techniques such as avoidance and spiritual strategies are cited commonly as means to deal with mental illnesses in India (Nadkarni et al., 2013a; Nadkarni, Murthy, Crome, & Rao, 2013b; Pereira et al., 2007).

Our findings have several implications. Notably, functional uses of alcohol such as inducing sleep or dealing with stress were cited by participants across all three categories that we examined. Whether as an instigator to begin drinking or a barrier to recovery, alcohol was frequently used as a coping mechanism. Thus, consistent with western societies, our results suggest that investment in interventions that emphasize healthier coping mechanisms would be useful for the prevention and treatment of AUD (Bowen et al., 2014; Brady & Sonne, 1999).

In contrast to many western societies (Wallhed Finn, Bakshi, & Andréasson, 2014), there was a persistent tendency to quantify alcohol abuse severity by physical rather than social consequences. Many of those with persistent AUD believed the consequences of drinking only extended to physical illness, which is consistent with existing studies whereby concerns about alcohol dependence were almost entirely attributed to fear of physical symptoms (Ghosh et al., 2012). Moreover, even for those who recovered from AUD, physical symptoms were both the prompt needed to seek help and a measure of their recovery status; many participants attributed recovery to absence of physical symptoms, such as no longer shaking or getting good sleep, regardless of drinking status. Simultaneously, there was generally little understanding of the societal consequences of AUD or the social changes involved in recovery. Together, the results suggest an inappropriate adoption of AUD as a physical illness without acknowledgement of surrounding social contexts. This could be exploited by developing psychosocial interventions integrated into physical health care platforms to encourage treatment-seeking behavior.

There were conflicting perspectives about the role of the doctor in treating AUD. In many with persistent AUD, the prevailing perspective was that the doctor can provide a quick recovery through medications. It appeared that by labeling AUD as an illness for which medical treatment is needed, many believed it would have a recovery process similar to other physical illnesses, with incremental progress and solutions external to the body, such as pills. Conversely, there were many recovered AUD participants who attributed their success to personal resolve, and many advised against a doctor. Thus, two perspectives emerged—that of the doctor as the only solution to an AUD and that of the doctor having no role. However, even in the former perspective, there was no psychosocial role for the doctor—an important component of AUD treatment (Huebner & Kantor, 2011). These understandings of the medical doctor are not compatible with a healing role. An understanding that the doctor’s role in the management of AUD falls between these two extremes and incorporates both medicines and psychosocial support is essential to encouraging better help-seeking behavior.

One of the limitations of our study is that the sample was entirely male. Although we interviewed only males to reflect the predominantly male sociodemographic of drinking and AUD in India, this limits the understanding of development and progression of AUD through a gendered lens. A second limitation is related to the method used to categorize the participants into the three groups. Participants were classified as persistent, recovered, or incident AUD according to the presence or absence of AUD at two timepoints, but this method is vulnerable to misclassification if the participant had relapsed and recovered frequently in between the two assessment points. This was evident in the interviews of some participants in both the recovered and persistent AUD group who described binge-drinking behavior. Nevertheless, the study’s limitations are offset by numerous strengths. Primarily, the detailed interviews of many participants allowed for a nuanced understanding of the natural history of AUD. Finally, this is one of the first community studies from India to assess long-term follow-up with detailed interviews examining the nature of development of and recovery from AUD.

In conclusion, this article offers a strong conceptualization and nuanced understanding of AUD across a spectrum of developmental courses. In doing so, it allows for identification of intervention targets for prevention and treatment of AUD in LMIC settings. On a broader scale, our findings add to the limited literature examining contextually specific conceptualizations of AUD.

## References

[CIT0001] Benegal V. (2005). India: Alcohol and public health. *Addiction*, 100, 1051–1056. doi:10.1111/j.1360-0443.2005.01176.x 16042631

[CIT0002] Bowen S., Witkiewitz K., Clifasefi S. L., Grow J., Chawla N., & Hsu S. (2014). Relative efficacy of mindfulness-based relapse prevention, standard relapse prevention, and treatment as usual for substance use disorders. *JAMA Psychiatry*, 71, 547–556. doi:10.1001/jamapsychiatry.2013.4546 24647726 PMC4489711

[CIT0003] Brady K. T., & Sonne S. C. (1999). The role of stress in alcohol use, alcoholism treatment, and relapse. *Alcohol Res Health*, 23, 263–271. 10890823 PMC6760383

[CIT0004] Braun V., & Clarke V. (2006). Using thematic analysis in psychology. *Qualitative Research in Psychology*, 3, 77–101. doi:10.1191/1478088706qp063oa

[CIT0032] Brennan P. L., Schutte K. K., & Moos R. H. (2005). Pain and use of alcohol to manage pain: Prevalence and 3-year outcomes among older problem and non-problem drinkers. *Addiction*, 100(6), 777–786. 15918808 10.1111/j.1360-0443.2005.01074.x

[CIT0033] Cutter H. S., Maloof B., Kurtz N. R., & Jones W. C. (1976). “Feeling no pain” differential responses to pain by alcoholics and nonalcoholics before and after drinking. *Journal of Studies on Alcohol*, 37(3), 273–277. 4661 10.15288/jsa.1976.37.273

[CIT0005] Eisenberg L. (1977). Disease and illness distinctions between professional and popular ideas of sickness. *Culture, Medicine and Psychiatry*, 1, 9–23. doi:10.1007/bf00114808 756356

[CIT0006] Finney J. W., & Moos R. (1991). The long-term course of treated alcoholism:I. Mortality, relapse and remission rates and comparisons with community controls. *Journal of Studies on Alcohol*, 52, 44–54. doi:10.15288/jsa.1991.52.44 1994122

[CIT0007] Gandhi A., Kumar C., Saha P., Sahoo B. K., & Sharma A. (2011). *India human development index: Towards social inclusion*. New Delhi, India: Oxford University Press.

[CIT0008] Ghosh S., Samanta A., & Mukherjee S. (2012). Patterns of alcohol consumption among male adults at a slum in Kolkata, India. *Journal of Health, Population and Nutrition*, 30, 73–81. doi:10.3329/jhpn.v30i1.11279 PMC3312362 22524122

[CIT0009] Government of India (2011). Census of India 2011.Government of India.

[CIT0010] Gual A., Lligona A., & Colom J. (1999). Five year outcome in alcohol dependence. A naturalistic study of 850 patients in Catalonia. *Alcohol and Alcoholism*, 34, 183–192. doi:10.1093/alcalc/34.2.183 10344779

[CIT0011] Hahn R. A., & Inhorn M. (2009). *Anthropology and public health: Bridging differences in culture and society*. Oxford, UK: Oxford University Press.

[CIT0012] Huebner R. B., & Kantor L. W. (2011). Advances in alcoholism treatment. *Alcohol Research and Health*, 33, 295–299. 23580014 PMC3860532

[CIT0013] Kar N., Sengupta S., Sharma P., & Rao G. (2003). Predictors of outcome following alcohol deaddiction treatment: A prospective longitudinal study for one year. *Indian Journal of Psychiatry*, 45, 174–177. 21206850 PMC2952164

[CIT0014] Kleinman A. (1980). *Patients and healers in the context of culture*. Berkeley, CA: University of California Press.

[CIT0034] Kuntsche E., Knibbe R., Gmel G., & Engels R. (2005). Why do young people drink? A review of drinking motives. *Clinical Psychology Review*, 25(7), 841–861. 16095785 10.1016/j.cpr.2005.06.002

[CIT0015] Kuruvilla P., & Jacob K. (2007). Five-year follow up for sobriety in a cohort of men who had attended an alcoholics anonymous programme in India. *National Medical Journal of India*, 20, 234–236. 18254518

[CIT0016] Legard R., Keegan J., & Ward K. (2003). In depth interviews In Ritchie J., Lewis J., Nicholls C. M., & Ormston R. (Eds.), *Qualitative research practice: A guide for social science students and researchers* (pp. 138–169). London, UK: Sage.

[CIT0017] Mohan D., Chopra A., & Sethi H. (2002). Incidence estimates of substance use disorders in a cohort from Delhi, India. *Indian Journal of Medical Research*, 115, 128–135. 12201177

[CIT0018] Nadkarni A., Dabholkar H., McCambridge J., Bhat B., Kumar S., Mohanraj R., Murthy P., & Patel V. (2013a). The explanatory models and coping strategies for alcohol use disorders: An exploratory qualitative study from India. *Asian Journal of Psychiatry*, 6, 521–527. doi:10.1016/j.ajp.2013.06.010 24309865 PMC3878642

[CIT0019] Nadkarni A., Murthy P., Crome I. B., & Rao R. (2013b). Alcohol use and alcohol-use disorders among older adults in India: A literature review. *Aging and Mental Health*, 17, 979–991. doi:10.1080/13607863.2013.793653 23659339

[CIT0020] Nadkarni A., Weiss H. A., Naik A., Bhat B., & Patel V. (2016). The six-year outcome of alcohol use disorders in men: A population based study from India. *Drug & Alcohol Dependence*, 162, 107–115. doi:10.1016/j.drugalcdep.2016.02.039 26994665 PMC4841788

[CIT0021] Napolitano D. A., & Jones C. (2006). Who needs “pukka1 anthropologists”? A study of the perceptions of the use of anthropology in tropical public health research. *Tropical Medicine and International Health*, 11, 1264–1275. 16903889 10.1111/j.1365-3156.2006.01669.x

[CIT0022] O’Connor A., & Daly J. (1985). Alcoholics. *A twenty year follow-up study. British Journal of Psychiatry*, 146, 645–647. doi:10.1192/bjp.146.6.645 4016479

[CIT0023] Patel V., Dourado R., De Souza N., & Dias Saxena F. (2001). *The state of Goa’s health 2001*. New Delhi, India: Voluntary Health Association of India.

[CIT0024] Pereira B., Andrew G., Pednekar S., Pai R., Pelto P., & Patel V. (2007). The explanatory models of depression in low income countries: Listening to women in India. *Journal of Affective Disorders*, 102, 209–218. doi:10.1016/j.jad.2006.09.025 17074394

[CIT0025] Perera B., & Torabi M. (2009). Motivations for alcohol use among men aged 16–30 years in Sri Lanka. *International Journal of Environmental Research and Public Health*, 6, 2408–2416. doi:10.3390/ijerph6092408 19826552 PMC2760418

[CIT0026] Pillai A., Nayak M., Greenfield T., Bond J., Nadkarni A., & Patel V. (2013). Patterns of alcohol use, their correlates, and impact in male drinkers: A population-based survey from Goa, India. *Social Psychiatry and Psychiatric Epidemiology*, 48, 275–282. 22752108 10.1007/s00127-012-0538-1 PMC3529206

[CIT0027] Rehm J., Mathers C., Popova S., Thavorncharoensap M., Teerawattananon Y., & Patra J. (2009). Global burden of disease and injury and economic cost attributable to alcohol use and alcohol-use disorders. *Lancet*, 373, 2223–2233. doi:10.1016/s0140-6736(09)60746-7 19560604

[CIT0028] Saunders J. B., Aasland O. G., Babor T. F., DeLaFuente J. R., & Grant M. (1993). Development of the alcohol use disorders identification test (AUDIT): WHO collaborative project on early detection of persons with harmful alcohol consumption II. *Addiction*, 88, 791–804. doi:10.1111/j.1360-0443.1993.tb02093.x 8329970

[CIT0029] Wallhed Finn S., Bakshi A.-S., & Andréasson S. (2014). Alcohol consumption, dependence, and treatment barriers: Perceptions among nontreatment seekers with alcohol dependence. *Substance Use and Misuse*, 49, 762–769. doi:10.3109/10826084.2014.891616 24601784

[CIT0030] Weiss M. G., & Somma D. (2007). Explanatory models in psychiatry In Dinesh Bhugra & Kamaldeep Bhui (Eds.), *Textbook of cultural psychiatry* (pp. 127–140). Cambridge, UK: Cambridge University Press.

[CIT0031] World Health Organisation (2014). *Global status report on alcohol and health 2014*. Geneva, Switzerland: World Health Organisation.

[CIT0035] Yan F. A., Beck K. H., Howard D., Shattuck T. D., & Kerr M. H. (2008). A structural model of alcohol use pathways among Latino youth. *American Journal of Health Behavior*, 32(2), 209–219. 18052861 10.5555/ajhb.2008.32.2.209

